# Prevalence, detection of virulence genes and antimicrobial susceptibility of *Escherichia coli* isolated from arbor acres broilers feeding cycle in China

**DOI:** 10.3389/fvets.2024.1500355

**Published:** 2024-11-28

**Authors:** Qian Zhou, Mengjun Tang, Xiaoyan Zhang, Xiujun Tang, Junxian Lu, Yushi Gao

**Affiliations:** Jiangsu Institute of Poultry Science, Yangzhou, China

**Keywords:** *Escherichia coli*, drug resistance, drug resistance gene, virulence gene, broiler feeding cycle

## Abstract

The prevalence of antimicrobial resistance originating from animals presents a significant threat to the treatment of animal disease, public health, and food safety. Researchers have focused on antibiotic resistance in *Escherichia coli* (*E. coli*), yet there are few reports on the resistance change during the feeding cycle. The purpose of this study was to investigate the prevalence and antibiotic resistance changes of *E. coli* in animal, environmental, and human samples during the broiler feeding cycle. Epidemiological surveys were performed in a farm with feeding AA broilers in Yangzhou, Jiangsu Province, China. Results showed that during a 42-days feeding cycle, 128 *E. coli* isolates were obtained from the cloaca of white-feathered broilers (*n* = 140), with an isolation rate of 91.4%, 27 *E. coli* isolates were obtained from Feed (*n* = 70) and 35 *E. coli* isolates were obtained from cage swabs (*n* = 70). A workers’ hands swabs sample isolation rate of 68.6% (24/35) was observed. Antibiotic susceptibility testing revealed that out of 214 *E. coli* isolates, varying degrees of resistance were observed against 14 antibiotics. Most strains were resistant to ampicillin, cephalothiophene, ciprofloxacin, tetracycline, sulfamisoxazole, sulfamethoxazole and florfenicol, with a resistance rate exceeding 80%. The resistant strains demonstrated relatively stable patterns in their resistance to various antibiotics. Of the six antibiotic resistance genes tested, the *floR* gene showed the highest detection rate (72.4%), followed by *qnrS* (43.0%), *mcr-1* (35.0%), *aadE-Sat4-aphA-3* (28.0%), *blaNDM* (8.4%), *aac(6′)-lb* (3.7%), and *cfr* (0). The highest detection rate for virulence genes was *yijp*. In summary, the isolation rate of *E. coli* and antibiotic resistance profile in broiler chickens remained stable throughout their feeding cycle. These findings can serve as a reference for the rational use of antibiotics in clinical settings, they can guide the use of veterinary drugs in poultry breeding.

## 1 Introduction

For a long time, the problem of drug resistance in animal bacteria has been of great concern (1). As a typical gram-negative bacteria, *Escherichia coli* (*E. coli*) was discovered by Escherich in 1885 (2), it usually colonized in the intestinal tract of warm-blooded animals, and is also widely prevalent in the gastrointestinal tracts, excreta, soil, water, and air of broiler chickens (3). In the healthy gastrointestinal tracts of chickens, the presence of some *E. coli* can maintain normal gut functions, forming a mutually beneficial relationship with the body (4). However, when immunity decreases or bacteria invade tissues outside the gut, they can trigger symptoms such as gastroenteritis (5). As the poultry industry continues to expand, the misuse and overuse of veterinary antibiotics have led to an emerging issue of antibiotic resistance (6–8). According to literature reports, the prevalence of *E. coli* in broiler chickens before their release for market increased from 28.33 to 90%, with antibiotic-resistant bacteria widely distributed in animals and the environment during breeding, leading to an overall increase in antimicrobial resistance (9). Liu et al. (10) conducted a large-scale epidemiological survey on chicken flocks in the Jiangsu region, revealing a high isolation rate of 99% (167/168) for *E. coli* isolates, with severe resistance to tetracycline, amoxicillin, sulfamethoxazole, and trimethoprim-sulfonamide. Similarly, Tong et al.’s findings indicate that more than 78% of *E. coli* isolates obtained from 18 provinces in China are resistant to tetracycline, doxycycline, and florfenicol (11). Ye et al. isolated extended-spectrum beta-lactamase-resistant *E. coli* from retail poultry products (12). Additionally, Wang et al. isolated tetracycline-resistant strains from both working environments in live poultry markets and workers’ hands swabs, with a *tet(X3)* resistance gene detection rate of 100% (13).

The proliferation of antibiotic-resistant strains and the expanding scope of their resistance are major contributors to the profound decline in antimicrobial efficacy (14). The increasing resistance among animal-origin bacteria also poses a significant threat to public health security (15). Antibiotic-resistant bacteria from animal sources can be transmitted to humans through the food chain by directly contacting animals carrying the resistant strain, consuming contaminated meat, eggs, milk, fruits, and vegetables, or drinking water contaminated with the resistant strain (16–18). If they are pathogenic bacteria, they may directly result in therapeutic failure for human diseases. And if they are non-pathogenic resistant bacteria, they could establish colonization in the gut and transfer their resistance genes to other pathogens, potentially causing other disease (19). In 2016, a study on *mcr-1* (mobilized colistin resistance) was published in the Lancet Infectious Diseases (20). This study revealed the discovery of *mcr-1* genes in *E. coli* for the first time, demonstrating a diverse host range. The researchers also isolated bacteria carrying *mcr-1* from animals, food, humans, and environments. These findings unveiled the complexity and diversity of their backgrounds, broad host range, and ability to coexist with other antibiotic-resistant genes. This poses a significant threat to current public health security. Furthermore, numerous studies have confirmed the impact of animal-source antibiotic resistance on human health and public health (21–23).

China is one of the largest consumers of chicken in the world, with poultry playing a significant role in Chinese cuisine (24, 25). Chicken breeds are primarily classified as either white-feathered broilers or yellow-feathered broilers. The white-feathered broiler has the fastest growth rate among all domestic fowl varieties and the lowest feed conversion ratio. Arbor Acres broilers (AA) are a representative breed of white-feathered chickens with a feeding cycle of 42 days, the first 3 weeks are the brooding period, followed by 4 to 6 weeks for fattening. At maturity, they can weigh up to 2.5 kilograms. It is exceptional meat performance characterized by high protein content, low fat content, and low caloric intake that is popular by consumers (26). Therefore, they are the preferred meat choice of McDonald’s, Kentucky Fried Chicken, and other fast-food companies.

In 2022, the National Health Commission, along with 12 other departments, jointly formulated the “National Action Plan for Controlling Antimicrobial Resistance (2022–2025),” aiming to curb the increasingly severe problem of antimicrobial resistance in China. *E. coli*, serving as a pivotal indicator for Gram-negative bacteria, is an important subject for real-time and accurate monitoring of bacterial sensitivity and resistance to antibiotics (27). Through *in vitro* detection of antibacterial drugs, the sensitivity and resistance of bacteria to drugs can be timely and accurately monitored (28). In previous reports, researchers had focused on antibiotic resistance in *E. coli*, yet there was little information available regarding resistance during the farming process. Traditional methods for comparing resistance merely judge the magnitude of resistance differences based on numerical values, without revealing their statistical significance, which may compromise the reliability of the data. In this study, we investigated a white-feathered broiler farm in Yangzhou City, focusing on the impact and changes in *E. coli* resistance from various perspectives (animals, environment, and humans), then evaluating the resistance genes and virulence genes presented in the isolated strains, examining the associations between the resistant phenotype and genotype by statistical analysis. The results of the study aim to provide references for controlling antimicrobial resistance and promoting animal health farming.

## 2 Materials and methods

### 2.1 Sample collection

The study was conducted on a broiler farm with feeding AA white feather broilers in Yangzhou, Jiangsu province, China. Before entering the new batch of chickens, the hen houses were disinfected three times in the vacancy period. A total of 300 chickens were observed, and adopted a cage feeding mode during 0 to 14 days with a density ranging of 15 chickens/m^2^ (a total of 10 cages with 2 m^2^/cage), a fence feeding mode during 15 to 42 days of 6 chickens/m^2^ (a total of two fences with 25 m^2^/fence), with one full-time worker in charge of the chicken shed and four assisting workers.

**Figure 1:**
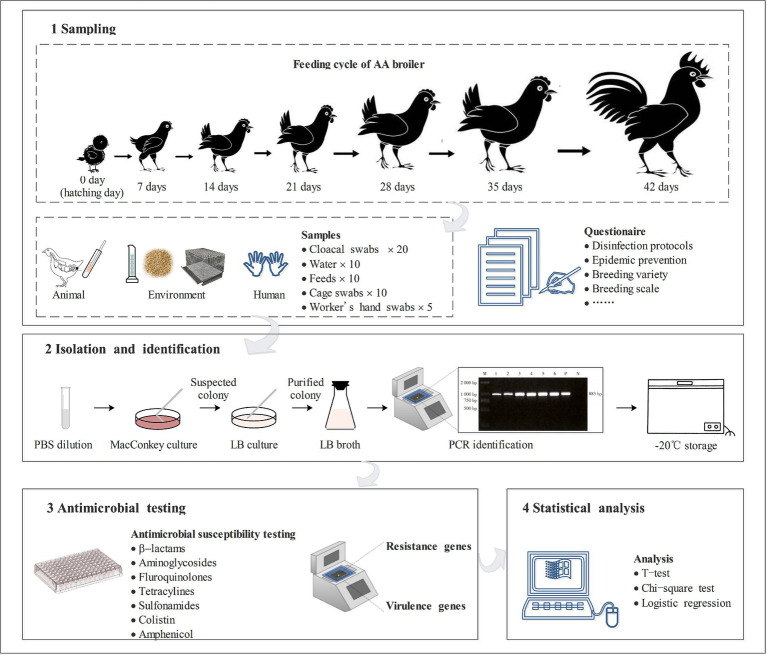
Schematic diagram overview of the study.

The sample collection was a longitudinal study. Animal samples (cloacal swabs), environmental samples (water, feed, and cage swabs), and human samples (workers’ hands swabs) were collected on 0 days (fetal feces), 7 days, 14 days, 21 days, 28 days, 35 days, and 42 days from February 19 to March 31, 2024. Twenty cloacal swabs samples were collected at each time point and stored them in Cary-Blair media (Oxoid Ltd., Hampshire, United Kingdom). Ten drinking nipples of chicken drinking water were sampled using sterile sampling tubes with 10 mL of water from 10 cages or fences. Ten feed samples (5 g) were randomly sampled from the through and mixed with 45 mL of tryptone soybean broth (Oxoid Ltd., Hampshire, UK). Cages’ swabs were randomly collected using sterile PBS (Takara Bio Inc., Dalian, China) cotton swabs. Workers’ hands swabs were collected by PBS cotton swabs after they finished the work of feeding and cleaning the chicken shed, the workers never wore gloves prior to and during the work before they were tested. 55 samples were collected at each time, a total of 385 samples were collected in seven sampling sessions. All samples were promptly placed in an ice box and transported to the laboratory within 6 h. A schematic diagram overview of this study was shown in Figure 1.

### 2.2 Feeding survey

This survey was a cross-sectional study. An unstructured questionnaire was administrated to the workers of the research farm at every sampling time. Through conversations with the workers to investigate the disease occurrences, disinfection protocols, vaccination practices, and drug use during the breeding cycle.

### 2.3 *Escherichia coli* isolation and identification

10 mL of water was centrifuged at 12000 rpm/min for 10 min and then suspended with 50 μL of PBS. 50 mL of a mix of feed and tryptone soybeans were preincubated at 37°C for 8 h. Cloacal swabs samples and cotton swabs samples (cages and workers) were diluted in 5 mL PBS. All samples were cultured in MacConkey culture medium (Haibo Biotech, Qingdao, China). Colonies were first subcultured onto MacConkey culture medium and then onto LB agar (Haibo Biotech) to obtain purified colonies. A single purified colony was selected to be incubated in LB broth (Haibo Biotech) for overnight suspension. All isolates were incubated at 37°C for 18 ~ 24 h. Isolates were stored in Brain Heart Infusion (BHI) broth (Oxoid, United Kingdom) containing 40% glycerol (Takara) at −20°C (1:1, V/V).

An aliquot of the overnight suspension culture (1 mL) was centrifuged and suspended in 500 μL PBS and washed twice. DNA from viable bacteria was extracted using the Rapid Bacterial Genomic DNA Isolation Kit (Sangon Biotech, Shanghai, China) following manufacturer’s guidelines. The quality of DNA was assessed by conventional agarose gel electrophoresis (1.5% w/v) and stored at −20°C for further analysis. *E. coli* strains were identified using PCR. Primer sequences were shown in Table 1. The reaction contained 1 μL template DNA, 12.5 μL Extaq premix (Takara Bio Inc., Kusatsu City, Japan), 1 μL forward primer (10 μmol/L), 1 μL reverse primer (10 μmol/L), and 9.5 μL ddH_2_O. GelRed stain (Biotium, Fremont, CA, United States) was added to 2 μL of PCR product and run on a 1.2% agarose gel for 40 min at 100 V. Agarose gels were visualized under UV light using a G: BOX imaging system (Syngene, Cambridge, United Kingdom). *E. coli* ATCC 25922 was used as a control strain.

**Table 1:** Sequences of primers and amplicon sizes.

Gene		Sequence(5′-3′)	Size (bp)	Annealing temperature (°C)
*E. coli*		F: ATGAAACGAGTTATTACCCTGTT	857	55
		R: CGCCAATAATCGATTGCACATT		
Amphenicol	*floR*	F: CACGTTGAGCCTCTATAT	868	55
		R: ATGCAGAAGTAGAACGCG		
β-lactams	*blaNDM*	F: GGTTTGGCGATCTGGTTTTC	621	53
		R: CGGAATGGCTCATCACGATC		
Colistin	*mcr-*1	F: ATGCCAGTTTCTTTCGCGTG	502	55
		R: TCGGCAAATTGCGCTTTTGGC		
Quinolones	*qnr*S	F: ACCTTCACCGCTTGCACATT	571	58
		R: CCAGTGCTTCGAGAATCAGT		
Multidrug resistance gene	*cfr*	F: GGGAGGATTTAATAAATAATTTTGGAGAAACAG	580	58
		R: CTTATATGTTCATCGAGTATATTCATTACCTCATC		
Quinolones	*aac(6′)-lb*	F: GGTTTGGCGATCTGGTTTTC	621	53
		R: CGGAATGGCTCATCACGATC		
Aminoglycosides	*aadE-Sat4-aphA-3*	F: GGAGAA ACTTTTGTCCACCTA CC	1,538	55
		R: AATGTCATACCACTTGTCCGC		
Adhesion-related genes	*tsh*	F: GTCTGTCAGACGTCTGTGTTTC	598	55
		R: ATAGGATGACAGGCTACCGAC		
	*fimC*	F: GCCGATGGTGTAAAGGATGG	475	
		R: GGGTAAGTGCGCCATAATCA		
	*mat*	F: CGACCTGGTCAGCAACAGCC	238	
		R: TCCACGCCCACATTCAGTGT		
Invasion and toxin-related genes	*ibeB*	F: GTTCTCACTCAGCCAGAACG	1,172	55
		R: CATCCAGCACTTCCAGATAAC		
	*vat*	F: TCCATGCTTCAACGTCTCAGAG	939	
		R: CTGTTGTCAGTGTCGTGAACG		
	*yijp*	F: TGGCTTGATTCTGCATCCGAT	517	
		R:CATCGTCTGCTGGTTGGTGAT		
	*ibeA*	F: GTATGACGGTGGGAACAAGAG	321	
		R: TGGCAATAGCAGCGGCAGTC		
Antiserum survial factor related genes	*ompA*	F: AGCTATCGCGATTGCAGTG	919	55
		R: GGTGTTGCCAGTAACCGG		
	*neuC*	F: GGTGGTACATTCCGGGATGTC	792	
		R: CATGGTGGTGAAAAGACATTAGC		
	*cvaC*	F: TCCAAGCGGACCCCTTATAG	598	
		R: CGCAGCATAGTTCCATGCT		
	*iss*	F: ATCACATAGGATTCTGCCG	309	
		CAGCGGAGTATAGATGCCA		
Iron transport related genes	*iroN*	F: CCTCCGACGATGATAATGAC	866	55
		R: GATACCATTATGCGTAATGCC		
	*fyuA*	F: ATGTGAAACTGCGTCTGGCG	728	
		R: CGCAGTAGGCACGATGTTGTA		
	*iucD*	F: GAAGCATATGACACAATCCTG	613	
		R: CAGAGTGAAGTCATCACGCAC		
	*irp2*	F: CTGATGAACTCACTCGCTATCC	440	
		R: AGCATCTCCTGGCTCTGCTC		
	*chuA*	F: GACGAACCAACGGTCAGGAT	278	
		R: TGCCGCCAGTACCAAAGACA		

### 2.4 Antimicrobial susceptibility testing

The susceptibility of *E. coli* isolates to antibiotics was determined by the broth microdilution method to test the minimum inhibitory concentrations (MICs), as recommended by the Technical Code of Practice of Antimicrobial Susceptibility Tests for Bacteria Isolated from Animals (NY/T 4142–2022). Bacteria were diluted to the density of the 0.5 McFarland standard (1.0 × 10^8^ CFU/mL) and inoculated into a drug sensitive plate (IN.KING, Tianjin, China) in a final bacterial suspension concentration of 5.0 × 10^5^ CFU/mL, with a quality control strain. Fourteen antibiotics from seven classes were tested: *β*-lactams (ampicillin, AMP; amoxicillin clavulanic acid, AM/CA; cephalothiophene, CEP; ceftiofur, CEF; meropenem, MEM), aminoglycosides (kanamycin, KAN; gentamicin, GM), fluroquinolones (ciprofloxacin, CIP), tetracylines (tetracycline, TET; doxycycline, DOX), sulfonamides (sulfamisoxazole, SIZ; sulfamethoxazole, SXT), colistin (pilumyxin E, COL) and amphenicol (florfenicol, FF). The resistance determination criteria are based on the Clinical and Laboratory Standards Institute (CLSI) 2020 guidelines (29), the MICs are categorized as resistant (Resistance, R), intermediate (Intermediate, I), or sensitive (Sensitivity, S; Supplementary Table S1). We calculated the total number of strains tested (N_total_), categorized the MICs into resistant (R), intermediate (I), and sensitive (S) groups, and separately counted the number of strains in each category (N_R_, N_I_, N_S_). Then we calculated the resistance rate (R%), intermediate sensitivity rate (I%), and sensitivity rate (S%) using the formulas N_R_/N_total_, N_I_/N_total_, and N_S_/N_total_, respectively. Strains resistant to 3 classes or more different antibiotics were classified as multidrug resistant (MDR) (30).

### 2.5 Detection of resistance genes and virulence genes

Isolates were assessed for the presence of resistance genes and virulence genes. Primer sequences and annealing temperature were shown in Table 1. The antibiotic resistance genes were *floR*, *blaNDM*, *mcr-1*, *qnrS*, *cfr*, *aac(6′)-lb*, and *aadE-Sat4-aphA-3*. And the virulence genes were adhesion-related genes (*tsh*, *fimC*, and *mat*), invasion and toxin-related genes (*ibeB*, *vat*, *yijp*, and *ibeA*), antiserum survial factor-related genes (*ompA*, *neuC*, *cvaC*, and *iss*) and iron transport-related genes (*iroN*, *fyuA*, *iucD*, *irp2*, and *chuA*) (31). PCR products were sequenced by Shanghai Bioengineering Co. (Shanghai, China), and the resulting sequences were compared against the GenBank database.

### 2.6 Statistical analysis

The data were inputted and arranged in WPS Office (version 10.9.2), Zhuhai Jinshan Office Software Co., Ltd. The hypothesis test (one sample test for a proportion) was employed to assess whether the isolation rate of *E. coli* on a specific day was different from the total isolation rate of the corresponding sample types. To investigate the association between antimicrobial resistance phenotype and genotype, an chi-square test of independence was conducted to determine whether there was a significant relationship between them. Subsequently, logistic regression analysis was performed to quantify the contribution of the resistance genes to antimicrobial resistance, with antimicrobial resistance (phenotype) as the dependent variable and the presence of resistance genes (genotype) as the independent variable. In the hypothesis test, we assumed an association between the prevalence of resistance and days, as well as an association between resistant phenotype and genotype. Statistical results with *p* < 0.05 indicated rejection of the hypothesis, suggesting a statistically significant association in both cases. The statistical analysis was carried out by SAS software (version 9.2), Institute Inc., Cary. The figures in this study were drawn using ChiPlot.^1^

## 3 Results

### 3.1 Feeding survey and isolation of *Escherichia coli* isolates

During the 42-days feeding cycle, the farm adopted a combination of initial cage rearing followed by fence feeding to demonstrate the actual production method of the AA broiler. One full-time worker was in charge of the hen house, however, there were four workers who participated in immunization, disinfection, and cleaning work. Routine feeding and vaccination were carried out without any additional interference with the growth of the chickens. The workers cleaned the ground and chute and disinfected on 29-days. China has already prohibited the use of antibiotics in animal feed, the well-being of chickens during the feeding cycle effectively guaranteed no therapeutic drugs were administered, thereby eliminating any potential interference caused by induced resistance.

As shown in Table 2, a total of 214 *E. coli* isolates were obtained, with a prevalence rate of 55.6%. The isolation rate from cloacal swabs was 91.4% (128/140), no *E. coli* isolates were detected in the drinking water, the detection rates for feed samples and cage swabs were 35.7% (27/70) and 50.0% (35/70), respectively. While that from workers’ hands was 68.6% (24/35), hands’ swabs from the full-time worker were positive for *E. coli* at each time point. The total isolation rates varied dynamically across different days, it reached a maximum of 70.9% on day 28 (*p* < 0.05), with a 100% isolation rate observed for isolates obtained from cloacal swabs on day 21. The highest isolation rates of feed and cage swabs were observed on day 28 (*p* < 0.05), which corresponded to the cleaning and disinfection on day 29. The workers’ hand swabs isolation rates fluctuated between 60 and 80%, statistical analysis did not reveal a significant difference (*p* > 0.05).

**Table 2:** *Escherichia coli* isolated from samples collected during feeding cycle.

Day	Animal	Environment			Human	Total
	Cloacal swabs	Water	Feed	Cage	Workers’ hands swabs	
Day 0	90.0% (18/20)	0 (0/10)	0 (0/10)^a^	10.0% (1/10)^a^	60.0% (3/5)	40.0% (22/55)^a^
Day 7	95.0% (19/20)	0 (0/10)	20.0% (2/10)^b^	40.0% (4/10)^b^	80.0% (4/5)	52.7% (29/55)^b^
Day 14	85.0% (17/20)	0 (0/10)	50.0% (5/10)^b^	50.0% (5/10)^b^	80.0% (4/5)	56.4% (31/55)^b^
Day 21	100% (20/20)	0 (0/10)	50.0% (5/10)^b^	60.0% (6/10)^b^	60.0% (3/5)	61.8% (34/55)^b^
Day 28	95.0% (19/20)	0 (0/10)	70.0% (7/10)^c^	100% (10/10)^c^	60.0% (3/5)	70.9% (39/55)^c^
Day 35	95.0% (19/20)	0 (0/10)	30.0% (3/10)^b^	40.0% (4/10)^b^	80.0% (4/5)	54.5% (30/55)^b^
Day 42	80.0% (16/20)	0 (0/10)	50.0% (5/10)^b^	50.0% (5/10)^b^	60.0% (3/5)	52.7% (29/55)^b^
Total	91.4% (128/140)	0 (0/70)	35.7% (27/70) ^b^	50.0% (35/70)^b^	68.6% (24/35)	55.6% (214/385)^b^

The same shoulder label letters or no shoulder label in the same column indicate no significant difference in results, whereas the difference is significant.

### 3.2 Antimicrobial resistance

**Figure 2:**
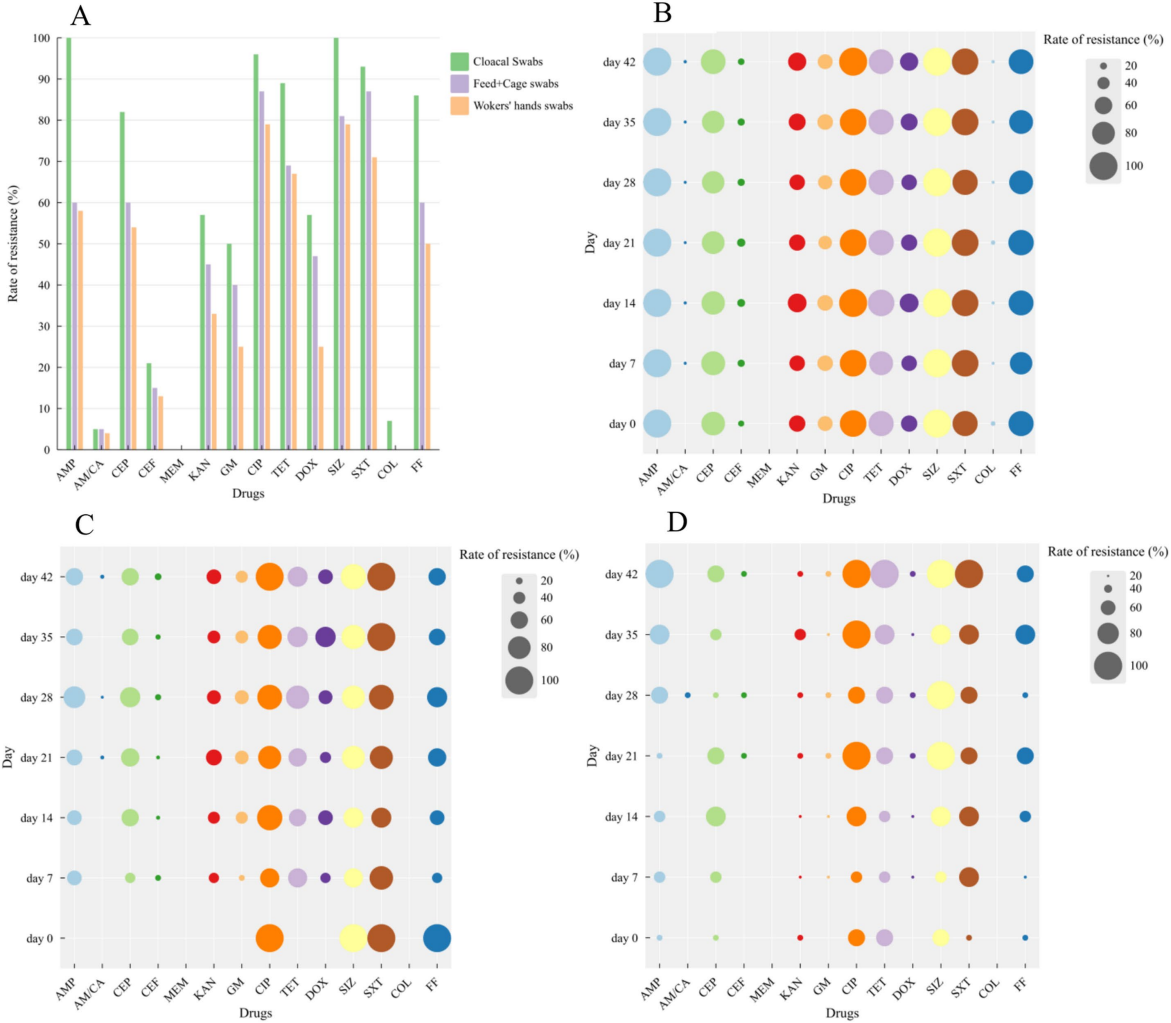
Resistance of *Escherichia coli* isolates (including cloacal swabs, Feeds, cage swabs and workers’ hands swabs) at different days to 14 kinds of antibiotics. (A) Bubble chart of drug resistance at different days isolated from cloacal swabs (B), Feeds and Cage swabs (C) and workers’ hands swabs (D).

The *E. coli* isolates displayed varying degrees of resistance to 14 antibiotics, as shown in Supplementary Tables S1–S3. In Figure 2A, the antimicrobial resistance rate of cloacal swabs isolates was significantly higher than that of environment isolates (feed and cage swabs) and workers’ hand swabs isolates (*p* < 0.05), except for AM/*CA.* The isolates exhibited high resistance against AMP, CEP, CIP, TET, SIZ, SXT, and FF, with a resistance rate exceeding 60%. They were moderately sensitive to CEF, KAN, GM, and DOX, with a resistance rate below 60%, thus making them suitable as alternative medications in the farming process. They were highly susceptible to AM/CA, MEM, and COL, with a resistance rate below 5%. The *E. coli* isolates showed significant different resistance to *β*-lactam antibiotics, with high resistance to AMP and CEP, while being sensitive to AM/CA, CEF, and MEM.

Bubble charts were used to analyze the antibiotic resistance profiles of *E. coli* isolates from different days, as shown in Figures 2B–D. Given that only one isolate was obtained from feed and cage swabs on day 0, the following results in susceptibility patterns were not considered this isolate. The isolated strains exhibited varying trends in resistance to five β-lactam antibiotics during the feeding cycle, AMP and CEP maintained high levels of resistance, while AM/CA and MEM showed stable sensitivity, and the resistance trend of CEP fluctuated dynamically. The isolates showed significant changes in sensitivity to two aminoglycosides (KAN and GM), but their trends were similar. Strains showed high-level resistance to CIP. For tetracycline and doxycycline, the isolates’ resistance rates were stable before day 21, after which they fluctuated dynamically and accompanied a decreasing resistance trend except for the isolates of workers’ hands swabs. Concerning sulfonamides, the isolates exhibited high-level resistance to SIZ and SXT, while they were highly sensitive to COL. During the breeding process, the resistant strains demonstrated relatively stable patterns in their resistance to various antibiotics.

### 3.3 Prevalence of antibiotic resistance genes and virulence genes

**Figure 3:**
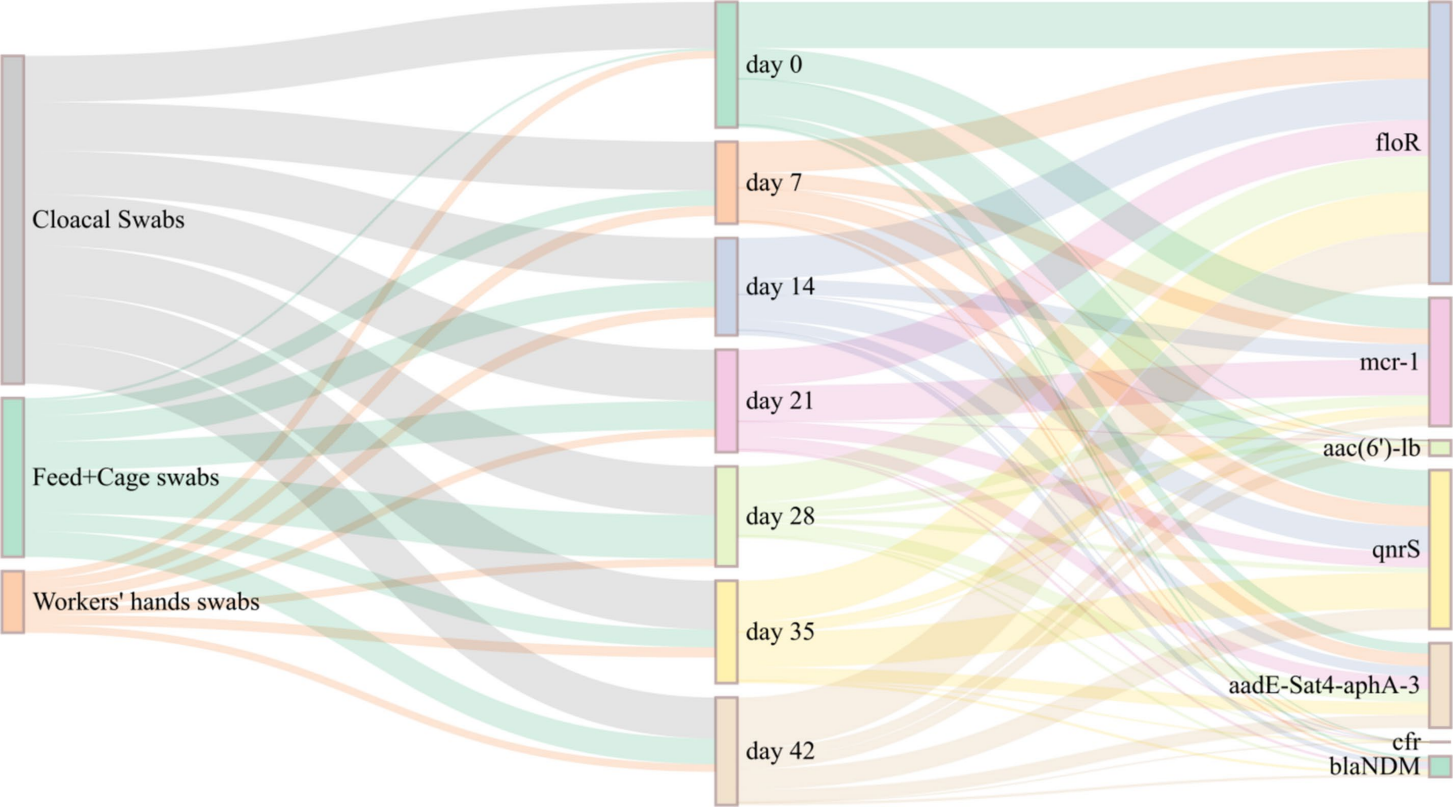
Sankey map of detection of drug resistance genes in *E. coli* isolates of different days.

A total of 214 *E. coli* isolates were examined for the presence of antibiotic resistance genes, including *floR*, *mcr-1*, *aac(6′)-lb*, *qnrS*, *aadE-Sat4-aphA-3*, *cfr*, and *blaNDM*. The *floR* gene exhibited the highest detection rate (72.4%), followed by *qnrS* (43.0%), *mcr-1* (35.0%), and *aadE-Sat4-aphA-3* (28.0%). The detection rates of *blaNDM* (8.4%) and *aac(6′)-lb* (3.7%) were lower. No detection of the multiple resistance gene *cfr* (0%). The Sankey map (Figure 3) indicates that the detection rates of different days of *E. coli* isolates for various antibiotic resistance genes varied. Among them, the *floR* gene had higher detection rates on days 0, 35, and 42. While the *mcr-1* gene had the highest detection rate on days 0 and 21. The detection rate of *qnrS* gene was highest on days 0 and 35. There was no discernible pattern in the detection of antibiotic resistance genes with changes in day.

**Figure 4:**
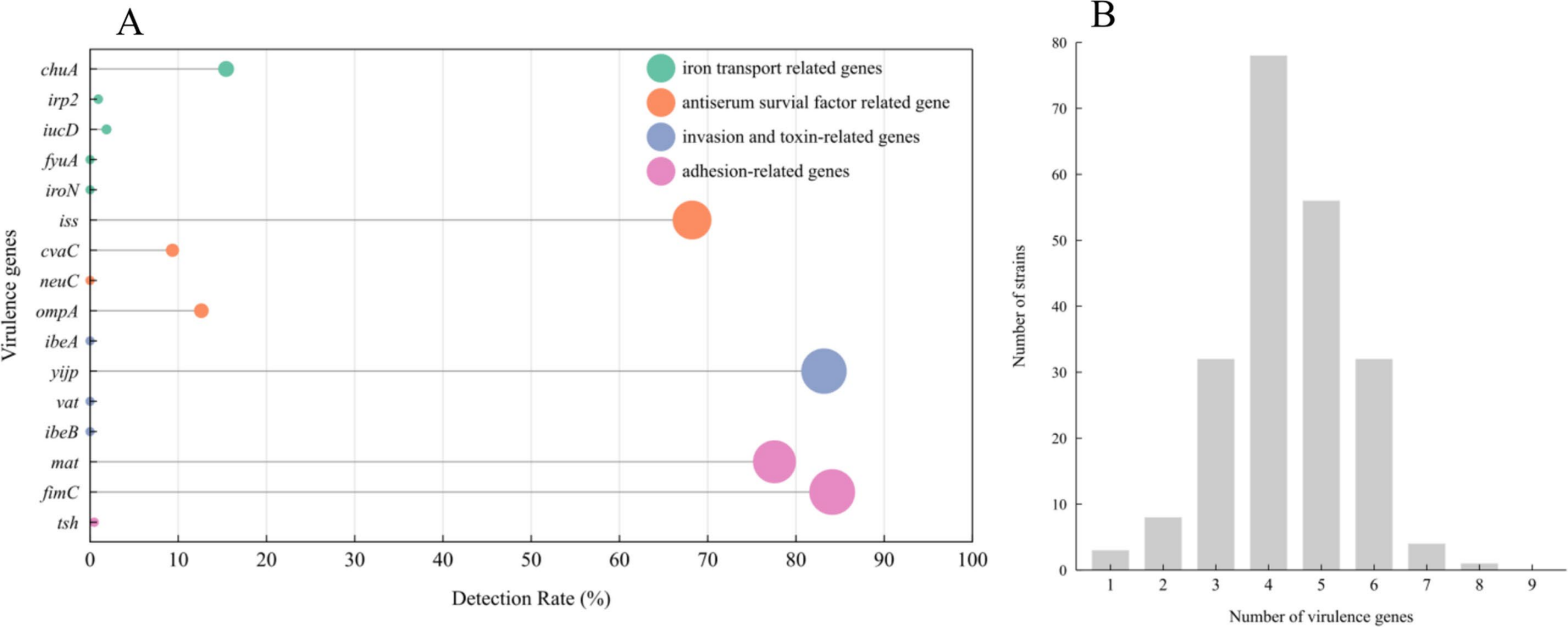
Distribution of *E. coli* virulence genes: 214 *E.coli* strains carrying 16 virulence genes (A). Number of virulence genes in *E. coli* isolates (B).

Among the 16 genes analyzed for virulence, *fimC*, *mat*, and *yijp* showed detection rates above 70%, *iss* had a detection rate of 68.2%, detection rates for *ompA* and *chuA* ranged between 10 to 20%, whereas *tsh*, *cvaC*, *iucD*, and *irp2* had detection rates below 10%. The remaining genes, namely *ibeB*, *vat*, *ibeA*, *neuC*, *iroN*, and *fyuA*, were not detected at all, as depicted in Figure 4A. As shown in Figure 4B, upon analysis of the number of virulence genes carried by various strains, it was discovered that 37 *E. coli* isolates harbored six or more virulence genes, accounting for 17.3%, 134 *E. coli* isolates carried four to five virulence genes, accounting for 62.6%, and 43 *E. coli* strain isolates carried three or fewer virulence genes, accounting for 20.1%.

### 3.4 Analysis of the correlation between antimicrobial phenotype and genotype

Due to the scarcity of environmental and human isolates, we focused on the correlation between drug resistance phenotype and genotype of animal-derived isolates. The statistical analysis as shown in Table 3, the result of chi-square test revealed a significant correlation (*P_χ2_* < 0.05) between the antimicrobial phenotypes and genotypes except for AMP and MEM. According to the parameter of β_1_, FF, KAN, and GM exhibited a β_1_ value greater than 0, whereas the other three drugs of AM/CA, CEP, and CEF possessed values less than 0. It indicated the respective contributions to the resistance rate in positive or negative terms: the detection of *floR* led to an increased resistance rate against FF, the detection of *aadE-Sat4-aphA-3* resulted in higher resistance rates against KAN and GM, and the negative correlation between *blaNDM* genes and antimicrobial resistance to AM/CA, CEP, and CEF. However, the P_model_ were not statistically significant, with *p* > 0.05, indicating that while resistance genes do indeed have an impact on the prevalence of resistance, this impact was not significantly pronounced from a statistical perspective.

**Table 3:** The chi-square test and logistic regression analysis of antimicrobial drugs and related genes.

Genes	Drugs	Drug resistance Number of strains carrying genes/Number of strains tested corresponding to category (ratios)			*P*_χ2_	Logistics	
		R	I	S		β_1_	*P*_logistics_
*floR*	FF	100/110 (90.9%)	3/5 (60%)	7/13 (53.8%)	<0.05	2.21	0.1554
*aadE-Sat4-aphA-3*	KAN	27/73 (37.0%)	1/5 (20%)	5/50 (10%)	<0.05	0.92	0.6904
	GM	23/64 (35.9%)	1/5 (20%)	9/59 (15.2%)	<0.05	0.87	0.7603
*blaNDM*	AMP	12/128 (9.4%)	0/0 (0)	0/0 (0)	—	—	—
	AM/CA	1/6 (16.7%)	1/5 (20%)	10/117 (8.5%)	<0.05	−8.17	0.1747
	CEP	2/105 (1.9%)	5/14 (35.7%)	5/9 (55.6%)	<0.05	−1.47	0.2095
	CEF	3/27 (11.1%)	1/5 (20%)	8/96 (8.3%)	<0.05	−5.07	0.3705
	MEM	0/0 (0)	0/0 (0)	12/128 (9.4%)	—	—	—
*qnrS*	CIP	69/123 (56.1%)	0/0 (0)	1/5 (20%)	<0.05	—	—
*aac(6′)-lb*	CIP	4/123 (3.2%)	0 (0)	2/5 (40%)	<0.05	—	—
*mcr-1*	COL	3/9 (33.3%)	/	47/119 (39.5%)	<0.05	—	—

“—” means that data did not conform to statistical testing requirements, statistical analysis was therefore infeasible.

## 4 Discussion

In this study, a total of 214 *E. coli* isolates were obtained during the AA white-feathered broiler rearing process. Of these, 128 isolates were obtained from cloacal swabs, 27 isolates were obtained from feed, and 35 isolates were obtained from cage swabs. 24 isolates were obtained from workers’ hand swabs. No *E. coli* was detected in the drinking water, which means that the drinking water system was hygienic and not contaminated by bacteria. The isolation rates reported in this study are generally consistent with those of other researchers in China, such as Li et al., who conducted a survey on the contamination of *E. coli* in three villages in Xinjiang’s Ili, with an isolation rate exceeding 93.8% (32); Lü et al. reported a separation rate of 86.98% for 430 samples collected from 30 large-scale poultry farms in Henan province (33); and Liu et al. found a high isolation rate of 99% in a survey of the prevalence of *E. coli* in nine poultry farms across Jiangsu Province (34). The results above indicate that the isolation rate of *E. coli* is relatively high in poultry farms.

Resistance phenotype revealed that *E. coli* isolates showed high resistance to AMP, CEF, CIP, TET, SIZ, and SXT. The resistance rate exceeded 80%. Conversely, they were highly susceptible to AM/CA, MEM, and COL. The resistance patterns of different days of *E. coli* isolates were generally consistent, with no significant changes in their antibiotic resistance profiles between cloacal swabs samples collected from different ages. This suggests that multidrug resistance in animal-origin *E. coli* demonstrates genetic stability, which can guide the precise and appropriate use of antibiotics in livestock and poultry farms based on antimicrobial resistance monitoring results during the early chicken stages. This is an important preventive measure for reducing the risk of zoonotic transmission. The emergence of antimicrobial resistance mainly arises from two routes: either via intrinsic genetic mutations, which are mostly transmitted vertically from parent to offspring (35), or through horizontally gene transfer (HGT) by mobile genetic elements (MGEs) such as transposons (36), plasmids (37), prophages (38), and integrons (39), allowing resistant genes to spread between and even across different strains, accelerating the emergence of multidrug-resistant strains. Additionally, MEM and COL are considered the “last line of defense” against gram-negative bacteria (40), and their use is prohibited in animal husbandry. No carbapenem-resistant Enterobacteriaceae (CRE) strains were detected in this study, indicating that the farm has not been affected by the spread of such resistant bacteria. Disparities in antimicrobial resistance are directly influenced by a variety of clinical scenarios. In a study by Zhang et al., antimicrobial resistance testing was conducted on eggs from Hebei Province, which revealed a high level of resistance to amoxicillin and sulfonamides in *E. coli*. The study also found that the formation of biofilms was associated with antimicrobial resistance (41). Zhao et al. identified and isolated *E. coli* strains from diseased chicken samples in the China Taizhou region, the results indicated a sensitivity rate of 67.7% to cefuroxime, making it a preferred drug for treatment (42). Gao et al. analyzed the antibiotic resistance patterns of *E. coli* in China Taiyuan and discovered a widespread occurrence of multi-drug resistance among isolates, the resistant genes carried by these isolates could potentially be transmitted inter-bacteria through conjugation (43). There have also been numerous reports in the international literature on antimicrobial resistant *E. coli*, Apostolakos et al. (44) found that Extended-spectrum *β*-lactamase (ESBL)- and plasmid mediated AmpC-type cephalosporinase (pAmpC)-producing *E. coli* (ESBL/pAmpC *E. coli*) in food-producing animals was prevalent, with substantial transfer between subsequent production levels. Yoon et al. (45) reported on *E. coli* that were highly resistant to fluoroquinolones. Messaili et al. (46) determined the virulence and antimicrobial resistance traits of 100 fecal *E. coli* strains isolated from clinically healthy chickens in Algeria, high resistance rates (62% ~ 97%) were noted for amoxicillin, amoxicillin/clavulanic acid, cefazolin, fluoroquinolones, tetracycline, trimethoprim, sulfonamides, and sulfamethoxazole/ trimethoprim, and 93% of strains were multidrug-resistant. Based on these findings, it is evident that the global situation of antibiotic resistance in *E. coli* is alarming. In terms of the differences in resistance, this may be attributed to factors such as the breed of broiler chickens, medication choice and dosage used in previous farms, feeding methods, and conditions.

Antimicrobial resistance is closely associated with the presence of resistant genes (47, 48). This study identified seven antibiotic resistant genes, with the *floR* gene detection rate reaching 72.4%, consistent with other research in China (49, 50). The *floR* gene is linked to flufenicol resistance, we observed that the resistance of flufenicol changes dynamically and the detection rate of the gene did not change correspondingly. This indicates that the *floR* gene is widely present in *E. coli*, functioning by encoding efflux pump proteins that pump fluoroquinolones out of the cell, thereby reducing antibiotic concentration to achieve bactericidal effects (34, 51). In contrast, no multiple antibiotic resistance gene *cfr* was detected in 152 *E. coli* isolates, possibly due to sampling from different days of broiler chickens but originating from the same source. Alternatively, this may be due to the gene’s current low prevalence in China, resulting in a lower overall detection rate, consistent with Zhao et al.’s findings (52). Since the discovery of *cfr* in various animal sources (chicken and pig) as well as in human isolates, *Staphylococcus aureus* and *Enterobacter aerogenes*, the emergence of this gene has led to the emergence of bacterial strains that may exhibit multidrug resistance to chloramphenicol, lincomycin, macrolides, streptogramins A, and oxazolidinones (53–55). Therefore, it is essential to continuously monitor this gene. Statistical analysis revealed a significant association (*P_χ2_* < 0.05) between the antimicrobial phenotypes and genotypes, the detection of *floR* and *aadE-Sat4-aphA-3* led to an increased resistance rate against their target drugs. However, the negative correlation between *blaNDM* genes and antimicrobial resistance to AM/CA, CEP, and CEF suggested that the *blaNDM* gene probably was not the target gene for these drugs. Therefore, it reminded us to integrate large-scale datasets through statistical analysis to investigate the correlation between resistance and other factors.

The highest detection rates for the virulence genes were *fimC*, *mat*, and *yijp*. These findings were partially consistent with the results of Ewers et al. (56) but contrasted with those of Hu et al. (57) and Aslam et al. (58). Hu et al. (57) reported that *fimC* (94.1) and *cvaC* (82.4%) had higher virulence genes detected in duck-origin pathogenic *E. coli*. Aslam et al. (19) found the majority of isolates (63%) harbored no virulence gene, only 11 (37%) isolates tested positive for two virulence genes. This suggests that differences in species may lead to differences in the distribution of virulence genes. The *fimC*, *mat*, and *yijp* genes correspond to adhesion-related genes and invasion and toxin-related genes, it implies that *fimC* and mat may be crucial factors for *E. coli* colonization in animal intestines, while *yijp* could potentially cause destructive effects. In Wang’s research, *E. coli* strains carrying three or more of the *tsh*, *cvaC*, *iss*, *iroN*, *iucD*, and *irp2* virulence genes were defined as pathogenic *E. coli* (59). This study identified three strains, two originating from animal cloacal swabs and one from an assisting workers’ hands swabs. These findings indicate potential public health risks and emphasize the importance of disinfection. Antibiotic-resistant bacteria ultimately have a profound impact on public health, manifesting in increased failure rates of infections to treatment, heightened severity of diseases, extended hospital stays, and, unfortunately, higher mortality rates. Borges et al. (60) found that *E. coli* recovered from poultry and extraintestinal pathogenic *E. coli* (ExPEC), responsible for most cases of urinary tract infection (UTI) and bloodstream infection (BSI) in humans, may share genetic characteristics, suggesting that poultry were a potential source of ExPEC. In the study of Jakobsen et al. (61), the cluster analysis of virulence gene and antimicrobial resistance profiles showed strong similarities between UTI patients, community-dwelling human isolates, meat, and production animal isolates, these strains from meat and production animals may pose a zoonotic risk.

## 5 Conclusion and recommendation

In summary, due to the increasing trend of antimicrobial resistance, it is necessary to address this issue at its roots in poultry husbandry. Intensive farming practices should be encouraged while reducing individual farming practices. Enhanced food safety inspections, improved breeding, and disease control conditions should be implemented. Given the stability of antimicrobial resistance, bacterial drug resistance monitoring can be carried out in the early stage of broiler breeding, allowing for prompt selection of sensitive and moderately susceptible drugs in case of disease outbreaks, thereby avoiding unnecessary and overuse of antibiotics. Rational and standardized drug usage should be strictly enforced, reducing the frequency of medication usage to minimize antimicrobial resistance. This is crucial to ensure the safety of animal products and to maintain human health.

## 6 Limitations of the study

This study examined the changes in resistance among broiler chickens during the breeding cycle, offering insight for the judicious application of medications in animal husbandry. However, the sampling was limited to a single breed, the environmental samples also only considered feed, drinking water, and cage, and did not consider other factors such as air and ground, which raises questions about the generalizability of the findings to other breeds, such as yellow-feathered broiler chickens. Therefore, this study can provide research ideas for the monitoring of drug resistance in other chicken breeding processes. In order to better understand the potential for antimicrobial transmission, future research should strive to expand sampling scope as far as possible, and employ molecular typing methods to observe the homology between animal isolates, environmental samples, and human isolates, in aid of tracing the source of infection, the route of transmission, and the colonization pattern.

## Data Availability

The raw data supporting the conclusions of this article will be made available by the authors, without undue reservation.

## Footnotes

^1^https://www.chiplot.online/
