# Supplementary material for: Prevalence, detection of virulence genes and antimicrobial susceptibility of Escherichia coli isolated from arbor acres broilers feeding cycle in China
Source: Front Vet Sci. 2024 Nov 28;11:1500355. doi: 10.3389/fvets.2024.1500355 (PMC11635991; doi:10.3389/fvets.2024.1500355)
Supplement: Supplementary file 1 [file Table_1.DOCX]

**Table S1 Criterion for determining antimicrobial resistance**

| Antimicrobial class | Drugs | MIC range, μg/mL | | |
| --- | --- | --- | --- | --- |
|  |  | R | I | S |
| β-lactams | AMP | ≧32 | 16 | ≦8 |
|  | AM/CA | ≧32/16 | 16/8 | ≦8/4 |
|  | CEP | ≧32 | 16 | ≦8 |
|  | CEF | ≧32 | 16 | ≦8 |
|  | MEM | ≧4 | 2 | ≦1 |
| Aminoglycosides | KAN | ≧64 | 32 | ≦16 |
|  | GM | ≧16 | 8 | ≦4 |
| Fluroquinolones | CIP | ≧1 | 0.5 | ≦0.25 |
| Tetracylines | TET | ≧16 | 8 | ≦4 |
|  | DOX | ≧16 | 8 | ≦4 |
| Sulfonamides | SIZ | ≧512 | / | ≦256 |
|  | SXT | ≧4/76 | / | ≦2/38 |
| Colistin | COL | ≧4 | / | ≦2 |
| Amphenicol | FF | ≧16 | 8 | ≦4 |

**Table S2 Antimicrobial resistance of *E. coli* isolates from cloacal swabs**

| Antimicrobial class | Drugs | Results of antimicrobial resistance | | | | | | |
| --- | --- | --- | --- | --- | --- | --- | --- | --- |
|  |  | N_total_ | N_R_ | N_I_ | N_S_ | R% | I% | S% |
| β-lactams | AMP | 128 | 128 | 0 | 0 | 100.0 | 0.0 | 0.0 |
|  | AM/CA | 128 | 6 | 5 | 117 | 4.7 | 3.9 | 91.4 |
|  | CEP | 128 | 105 | 14 | 9 | 82.0 | 10.9 | 7.0 |
|  | CEF | 128 | 27 | 5 | 96 | 21.1 | 3.9 | 75.0 |
|  | MEM | 128 | 0 | 0 | 128 | 0.0 | 0.0 | 100.0 |
| Aminoglycosides | KAN | 128 | 73 | 5 | 50 | 57.0 | 3.9 | 39.1 |
|  | GM | 128 | 64 | 5 | 59 | 50.0 | 3.9 | 46.1 |
| Fluroquinolones | CIP | 128 | 123 | 0 | 5 | 96.1 | 0.0 | 3.9 |
| Tetracylines | TET | 128 | 114 | 2 | 12 | 89.1 | 1.6 | 9.4 |
|  | DOX | 128 | 73 | 34 | 21 | 57.0 | 26.6 | 16.4 |
| Sulfonamides | SIZ | 128 | 128 | 0 | 0 | 100.0 | 0.0 | 0.0 |
|  | SXT | 128 | 119 | 7 | 2 | 93.0 | 5.5 | 1.6 |
| Colistin | COL | 128 | 9 | 0 | 119 | 7.0 | 0.0 | 93.0 |
| Amphenicol | FF | 128 | 110 | 5 | 13 | 85.9 | 3.9 | 10.2 |

**Table S3 Antimicrobial resistance of *E. coli* isolates from feed and cage swabs**

| Antimicrobial class | Drugs | Results of antimicrobial resistance | | | | | | |
| --- | --- | --- | --- | --- | --- | --- | --- | --- |
|  |  | N_total_ | N_R_ | N_I_ | N_S_ | R% | I% | S% |
| β-lactams | AMP | 62 | 37 | 21 | 4 | 59.7 | 33.9 | 6.5 |
|  | AM/CA | 62 | 3 | 45 | 14 | 4.8 | 72.6 | 22.6 |
|  | CEP | 62 | 37 | 14 | 11 | 59.7 | 22.6 | 17.7 |
|  | CEF | 62 | 9 | 13 | 40 | 14.5 | 21.0 | 64.5 |
|  | MEM | 62 | 0 | 0 | 62 | 0.0 | 0.0 | 100.0 |
| Aminoglycosides | KAN | 62 | 28 | 16 | 18 | 45.2 | 25.8 | 29.0 |
|  | GM | 62 | 25 | 25 | 12 | 40.3 | 40.3 | 19.4 |
| Fluroquinolones | CIP | 62 | 54 | 5 | 3 | 87.1 | 8.1 | 4.8 |
| Tetracylines | TET | 62 | 43 | 10 | 9 | 69.4 | 16.1 | 14.5 |
|  | DOX | 62 | 29 | 12 | 21 | 46.8 | 19.4 | 33.9 |
| Sulfonamides | SIZ | 62 | 50 | 0 | 12 | 80.6 | 0.0 | 19.4 |
|  | SXT | 62 | 54 | 0 | 8 | 87.1 | 0.0 | 12.9 |
| Colistin | COL | 62 | 0 | 0 | 62 | 0.0 | 0.0 | 100.0 |
| Amphenicol | FF | 62 | 37 | 18 | 7 | 59.7 | 29.0 | 11.3 |

**Table S4 Antimicrobial resistance of *E. coli* isolates from workers’ hands swabs**

| Antimicrobial class | Drugs | Results of antimicrobial resistance | | | | | | |
| --- | --- | --- | --- | --- | --- | --- | --- | --- |
|  |  | N_total_ | N_R_ | N_I_ | N_S_ | R% | I% | S% |
| β-lactams | AMP | 24 | 14 | 7 | 3 | 58.3 | 29.2 | 12.5 |
|  | AM/CA | 24 | 1 | 18 | 5 | 4.2 | 75.0 | 20.8 |
|  | CEP | 24 | 13 | 5 | 6 | 54.2 | 20.8 | 25.0 |
|  | CEF | 24 | 3 | 17 | 4 | 12.5 | 70.8 | 16.7 |
|  | MEM | 24 | 0 | 0 | 24 | 0.0 | 0.0 | 100.0 |
| Aminoglycosides | KAN | 24 | 8 | 3 | 13 | 33.3 | 12.5 | 54.2 |
|  | GM | 24 | 6 | 3 | 15 | 25.0 | 12.5 | 62.5 |
| Fluroquinolones | CIP | 24 | 19 | 2 | 3 | 79.2 | 8.3 | 12.5 |
| Tetracylines | TET | 24 | 16 | 3 | 5 | 66.7 | 12.5 | 20.8 |
|  | DOX | 24 | 6 | 12 | 6 | 25.0 | 50.0 | 25.0 |
| Sulfonamides | SIZ | 24 | 19 | 0 | 5 | 79.2 | 0.0 | 20.8 |
|  | SXT | 24 | 17 | 0 | 7 | 70.8 | 0.0 | 29.2 |
| Colistin | COL | 24 | 0 | 0 | 24 | 0.0 | 0.0 | 100.0 |
| Amphenicol | FF | 24 | 12 | 4 | 8 | 50.0 | 16.7 | 33.3 |
